# Case Report: Double-click on the ventricle: ventricular safety pacing triggered by right atrial lead dislodgement in a patient after right atrial tumor resection

**DOI:** 10.3389/fcvm.2026.1903535

**Published:** 2026-09-21

**Authors:** Xiaohan Qin, Jingbo Fan, Taibo Chen, Deyan Yang

**Affiliations:** 1 Department of Cardiology, Peking Union Medical College Hospital, Chinese Academy of Medical Science and Peking Union Medical College, Beijing, China; 2 Department of Internal Medicine, Peking Union Medical College Hospital, Chinese Academy of Medical Science and Peking Union Medical College, Beijing, China

**Keywords:** lead dislodgement, pacemaker, right atrial tumor resection, ventricular capture, ventricular safety pacing

## Abstract

**Background:**

Atrial lead dislodgment is not uncommon after permanent pacemaker implantation, especially in patients with atrial anatomic abnormity. Unique electrocardiography manifestation might give clues for promptly diagnosis. In patients with reconstructed right atrium, optimal pacing strategies and lead stability remain challenging.

**Case presentation:**

A 44-year-old woman developed symptomatic sinus node dysfunction after right atrial tumor resection and reconstruction. A dual-chamber pacemaker was implanted, resulting in symptom resolution. At routine follow-up, electrocardiography showed ventricle was intermittently captured by one initial pacing artifact which was immediately followed by another pacing artifact at a fixed interval of 110-ms, consistent with ventricular safety pacing. Device interrogation revealed normal ventricular lead parameters but loss of atrial capture. Imaging confirmed atrial lead dislodgement into the right ventricle. The atrial lead was subsequently extracted and the device was reprogrammed to VVI mode. After 3 months follow-up, the patient remained asymptomatic and was with normal cardiac function.

**Conclusion:**

This case highlights the importance of diagnostic role of electrocardiography to detect lead dislodgement. In patents with surgically altered right atrium, individualized pacing strategies, such as single-chamber ventricular pacing, may be appropriate. Novel atrial pacing lead with fix helix or leadless pacing might do some help to reduce the risk of dislodgement.

## 1 Introduction

Sinus node dysfunction (SND) is a common complication following cardiac surgery, especially when surgical manipulation involves the right atrium (RA) or the sinoatrial node region. The underlying mechanisms are multifactorial and include direct surgical injury, ischemia, inflammation, etc. (1). Although postoperative bradyarrhythmia is often transient, persistent or delayed symptomatic SND may require permanent pacemaker implantation (2).

Current ESC and ACC/AHA/HRS guidelines recommend permanent pacing for patients with symptomatic SND, with atrial-based pacing generally preferred when atrioventricular conduction is preserved because it maintains AV synchrony and may reduce the long-term risk of atrial arrhythmias and pacing-induced ventricular dysfunction (3–5). However, these guidelines provide limited guidance for patients with complex postoperative anatomy, particularly those undergoing extensive RA resection and reconstruction.

Lead-related complications are the leading cause of reintervention after pacemaker implantation. Atrial lead position was an independent predictor of lead dislodgement (6). In patients with surgically reconstructed RA, atrial lead implantation may be particularly challenging because of altered anatomy, atrial scarring, patch reconstruction, and reduced availability of viable myocardium for stable lead fixation. These factors may increase the risk of lead instability and complicate long-term pacing management.

During routine follow-up, pacemaker malfunction may be recognized on ECG by failure of capture, sensing abnormalities, loss of atrioventricular synchrony, unexpected pacing artifacts, or changes in paced QRS morphology (7). Although atrial lead dislodgement most commonly presents with loss of capture or abnormal pacing parameters, rare ECG manifestations may occur when a displaced lead interacts with adjacent cardiac structures, creating diagnostic uncertainty.

Here, we report a case of atrial lead dislodgement into the right ventricle (RV) causing atrial pacing-induced ventricular capture and subsequent ventricular safety pacing (VSP) in a patient with reconstructed RA after tumor resection. This case highlights the diagnostic value of careful ECG interpretation during routine device follow-up and discusses pacing strategies in patients with surgically reconstructed RA.

## 2 Case description

A 44-year-old woman with no prior history of arrhythmia was incidentally found to have a RA mass during a routine health examination in 2019. Despite two transcatheter embolization procedures targeting the presumed feeding vessels between 2019 and 2020, follow-up imaging demonstrated persistent tumor size. In April 2025, she presented with palpitations and lower-extremity edema. Preoperative Holter monitoring showed frequent premature atrial contractions, short runs of atrial tachycardia, and occasional premature ventricular contractions, without significant bradyarrhythmia. Repeat imaging demonstrated a persistent RA mass measuring approximately 38 mm × 30 mm (Figure 1A). In May, 2025, complete surgical tumor resection was performed under cardiopulmonary bypass. Intraoperatively, the tumor was found to be located outside the RA, extending from the superior vena cava junction superiorly to the inferior vena cava junction inferiorly, with posterior extension along the interatrial groove. The mass was well encapsulated and clearly demarcated from the surrounding tissues. It was excised *en bloc* together with the adjacent RA free wall, and the resultant atrial defect was reconstructed using a bovine pericardial patch. Histopathological examination confirmed paraganglioma.

**Figure 1:**
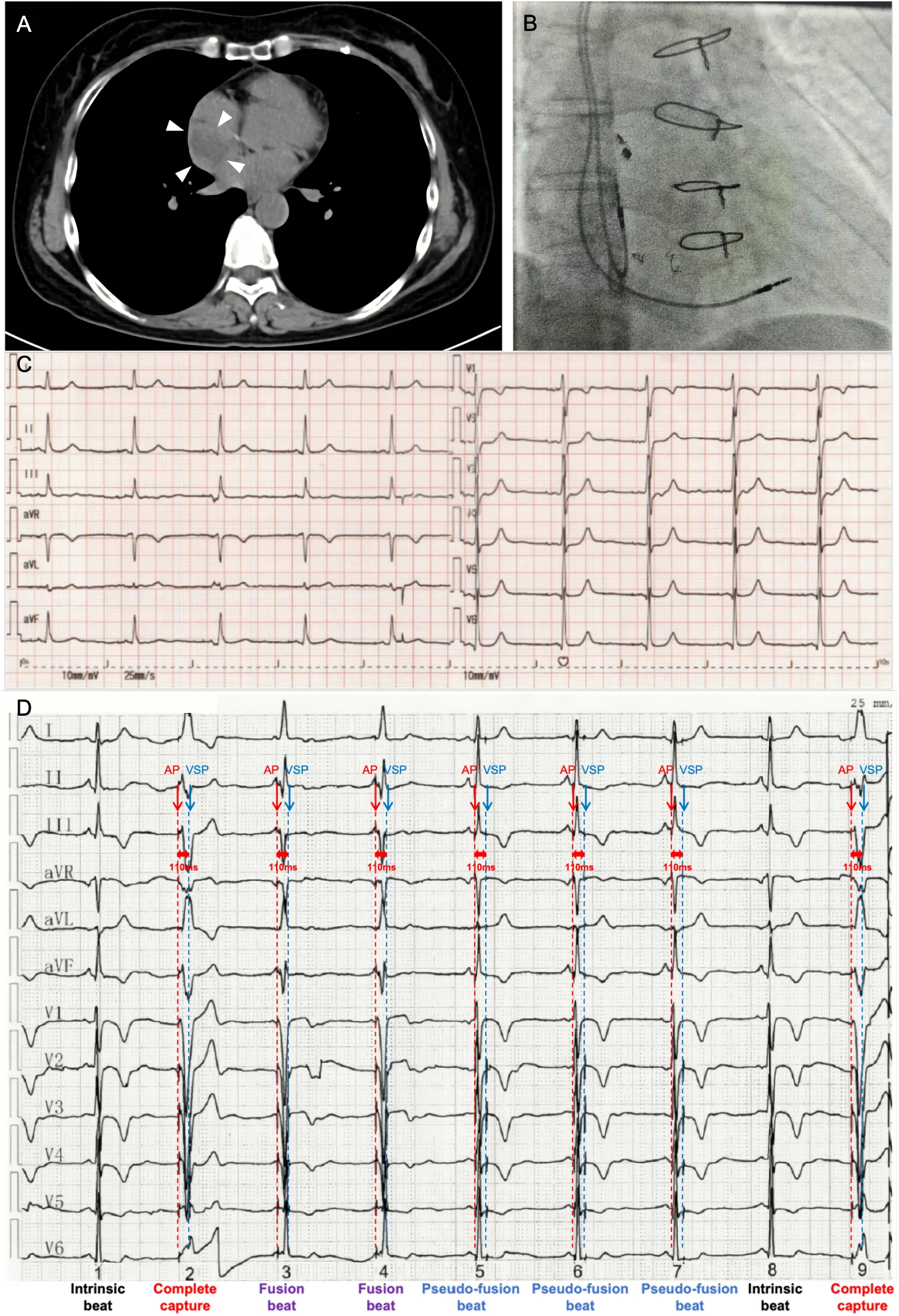
Pacemaker implantation, baseline pacing configuration, and electrocardiographic manifestation of atrial lead dislodgement. **(A)** A soft tissue mass indicated by white arrow in pre-operative chest CT (axial view). **(B)** Fluoroscopic image obtained during dual-chamber pacemaker implantation. The atrial lead (Medtronic 5076-52MR) was positioned in the right atrial appendage, and the ventricular lead (Medtronic 5076-58MRI) was implanted in the right ventricular septum. **(C)** The 12-lead electrocardiogram recorded immediately after pacemaker implantation. The device was programmed in AAI–DDD mode. Atrial pacing with intact intrinsic atrioventricular conduction resulted in atrial-paced ventricular-sensed rhythm (AP-VS), demonstrating appropriate atrial capture and normal ventricular activation through the native conduction system. **(D)** The 12-lead ECG obtained at routine follow-up 3 months after implantation at presentation with number marker. On the 12-lead ECG, intrinsic sinus P waves could be seen preceding QRS complexes (No. 1, 3, 4, 5, 6, 7, and 8), which indicated baseline sinus rhythm. QRS complexes No. 1 and 8 showed intrinsic QRS waves activated by intrinsic sinus P waves through the native conduction system without pacing delivery. Ventricle was captured on QRS complexes No. 2, 3, 4 and 9. QRS pattern of No. 2 and 9 documented totally capture. Those of No. 3 and 4 indicated fusion with QRS pattern being a hybrid of spontaneous and totally paced beats. QRS pattern of No. 5, 6 and 7 indicated pseudo-fusion with identical QRS morphology to those of No. 1 and 8. AP (red arrow), atrial pacing; VSP (blue arrow), ventricular safety pacing.

Early postoperative ECG recorded junctional escape rhythm with a heart rate (HR) of approximately 48 bpm. Over the following months, progressive sinus bradycardia developed, with resting HR decreasing to approximately 35 bpm without any symptom. Holter monitoring in September 2025 revealed sinus bradycardia ranging from 32 to 91 bpm (mean 47 bpm), 46 sinus pauses exceeding 2 s, with a maximum RR interval of 3.84 s. In October 2025, the patient developed postural dizziness and intermittent chest discomfort, leading to a diagnosis of sick sinus syndrome (SSS).

In November 2025, a dual-chamber permanent pacemaker (Medtronic Astra XT DR MRI X2DR01) was implanted via left axillary vein, with the atrial lead (5076-52MR) positioned in the RA appendage and the ventricular lead (5076-58MRI) in the RV septum (Figure 1B). Intraoperative measurements showed a slightly higher atrial capture threshold as 2.0 V at 0.4 ms (Table 1). The device was programmed in AAI–DDD mode with a lower rate limit of 60 bpm, a paced atrioventricular delay of 180-ms, and a sensed atrioventricular delay of 150-ms. Postoperative electrocardiography demonstrated appropriate atrial pacing with ventricular sensing (AP-VS) (Figure 1C), and echocardiography confirmed preserved left ventricular ejection fraction of 62%.

**Table 1:** Pacemaker interrogation parameters immediate post-implantation and 3 months post-operation.

	At the time of implantation			3-month post-operation		
Lead position	Capture threshold	Sensing amplitude	Impedance	Capture threshold	Sensing amplitude	Impedance
RA	2.0 V@0.4 ms	4.0 mV	750 Ω	/	5.1 mV	437 Ω
RV	0.7 V@0.4 ms	8.6 mV	754 Ω	0.75 V@0.4 ms	7.5 mV	513 Ω

At routine 3-month follow-up, the patient remained asymptomatic. Device interrogation revealed a high pacing burden (AP 98.3%, VP 92.3%) and loss of RA capture, whereas ventricular lead parameters remained stable (Table 1). A 12-lead ECG demonstrated ventricular activation immediately after atrial pacing stimulus, followed by a second pacing spike at a fixed 110-ms interval (Figure 1D). This pattern was consistent with VSP. VSP is a protective algorithm designed to prevent inappropriately inhibition of ventricular pacing that could lead to ventricular asystole when the ventricular lead mistakenly senses an atrial pacing artifact as a native ventricular event. In the Medtronic system, atrial pacing initiates a 110-ms crosstalk sensing window, including 30-ms post-atrial ventricular blanking period. If the ventricular lead senses any electrical event—whether an atrial pacing artifact, an intrinsic ventricular depolarization, or the signals—during this window, VSP is delivered at the window's end. In this ECG, two adjacent pacing artifacts could be seen (Figure 1D, No. 5–7). The second pacing artifacts followed the first ones at exactly 110 ms. An uncommon phenomenon of the present ECG was that RV was directly captured by atrial pacing. The subsequent ventricular depolarization was sensed by the ventricular lead within the programmed crosstalk sensing window, thereby triggering VSP. Although VSP was appropriately delivered, the right ventricular myocardium had already been depolarized by the displaced atrial lead and remained within its effective refractory period. Consequently, the VSP stimulus failed to recapture the ventricle. Laboratory testing, including complete blood count and inflammatory markers was unremarkable, and no structural or functional abnormalities were found by transthoracic echocardiography. Intracardiac electrograms demonstrated atrial pacing followed by ventricular sensing and VSP. Immediately after each atrial pacing stimulus, ventricular activation was recorded without an intervening atrial electrogram, indicating direct ventricular capture by the atrial lead (Figure 2C). These findings, together with chest radiography (Figures 2A,B), confirmed displacement of the atrial lead into the RV.

**Figure 2:**
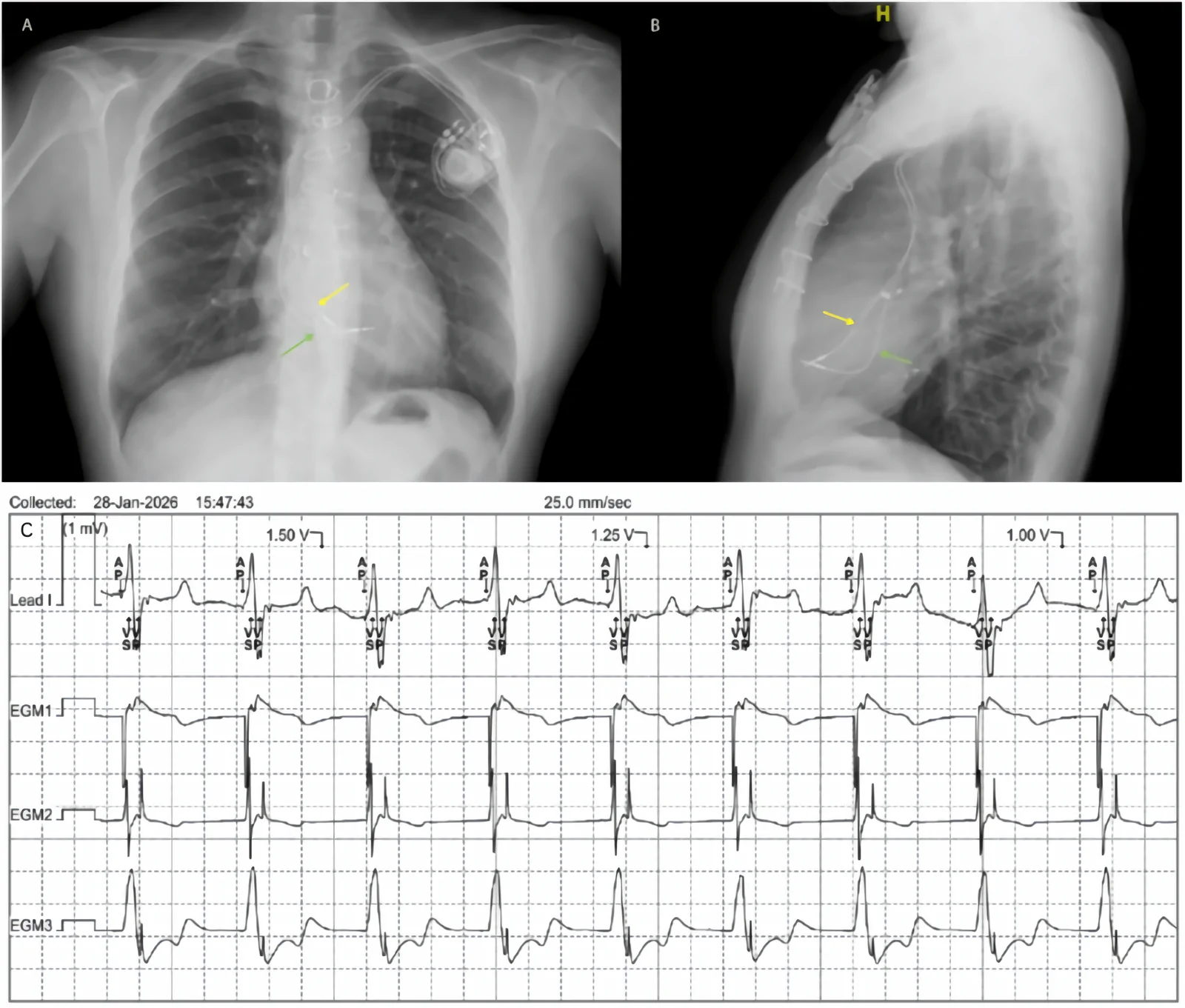
Chest x-ray showed right ventricular lead (green arrows) was in the interventricular septum and right atrial lead (yellow arrows) was dislodged in the tricuspid valve annulus. **(A)** Posterior-anterior view. **(B)** Lateral view. **(C)** Intracardiac electrogram (EGM). Showed from top to bottom are the surface ECG with marker, EGM1 source of A-tip to Aring, EGM2 source of RV-tip to RV-ring and EGM3 source of Can to RV-ring. AP, atrial pacing; VP, ventricular pacing; VS, ventricular sensing.

Given the surgically reconstructed RA and high risk of recurrent lead instability, the pacemaker was reprogrammed to VVI mode, which immediately resolved the abnormal pacing pattern. The dislodged atrial lead was subsequently extracted in February, 2026 after multidisciplinary evaluation and shared decision-making. At 3-month follow-up in May 2026, the patient remained asymptomatic. Device interrogation demonstrated stable RV lead function with a pacing percentage of 87.2%, sensing amplitude of 6.5 mV, impedance of 437 Ω, and capture threshold of 0.75 V at 0.4 ms. Transthoracic echocardiography showed preserved biventricular systolic function without ventricular remodeling. Holter monitoring revealed a stable paced rhythm with a mean HR of 65 bpm (range 59–125 bpm), no pauses exceeding 2 s, no atrial fibrillation, and no clinically significant ventricular arrhythmias. Despite the relatively high ventricular pacing percentage, no evidence of pacemaker syndrome or pacing-induced cardiomyopathy was observed. Continued VVI pacing and close follow-up were therefore recommended.

## 3 Discussion

### 3.1 Importance of ECG manifestation during device follow-up

Lead dislodgement may remain clinically silent, particularly in patients with preserved intrinsic rhythm or a functioning ventricular lead (8). Classical electrocardiographic manifestations include loss of capture, pacing spikes without subsequent depolarization, abrupt changes in paced QRS morphology, undersensing, oversensing, and intermittent capture. However, atrial lead dislodgement may occasionally produce more complex ECG patterns depending on its final location. Previous reports have described alternating QRS morphologies, ventricular fusion or pseudo-fusion beats, and RV outflow tract capture caused by chronic atrial lead dislodgement. Singh et al. had reported RV lead dislodgement in a patient with dual chamber pacemaker, where the ventricular lead in RA sensed atrial paced P wave during cross-talk window and triggered persistent VSP that failed to capture the RV (9). The combination of atrial pacing-induced ventricular capture and VSP observed in our patient is uncommon and may easily be misinterpreted as routine dual-chamber pacing unless marker-channel recordings and intracardiac electrograms are carefully analyzed.

Two closely spaced pacing artifacts on surface ECG may have several other potential mechanisms except for VSP. First, when both artifacts represent ventricular outputs, possible mechanisms include sequential left and right ventricular pacing during cardiac resynchronization therapy, or conduction system pacing (His bundle pacing or left bundle branch area pacing) with a backup ventricular pacing output (10–13). Second, one spike may represent atrial pacing and the other ventricular pacing during atrioventricular sequential pacing with a deliberately short-programmed AV delay. This pattern is most commonly encountered in dual-chamber pacing for hypertrophic obstructive cardiomyopathy to reduce left ventricular outflow tract obstruction, or in patients undergoing left bundle branch area pacing, where the programmed AV delay is intentionally shortened relative to the intrinsic AV conduction to achieve optimal fusion with native right bundle branch conduction (14, 15). In the present case, these alternative diagnoses were excluded because the patient had a conventional dual-chamber pacemaker without a coronary sinus or conduction system pacing lead, the programmed paced AV delay was 180 ms. Therefore, interpretation requires integration of device type, lead configuration, programmed parameters, intracardiac electrograms, and imaging findings.

Furthermore, the fixed 110-ms interval between the two pacing artifacts in this patient precisely matched the manufacturer-specific VSP interval of the implanted Medtronic device. VSP algorithms are designed to prevent inappropriate inhibition of ventricular pacing. After an atrial pacing stimulus, the ventricular channel enters a blanking period followed by a crosstalk sensing window. If a ventricular event is sensed during this window, the device delivers a VSP output at a manufacturer-specific interval rather than waiting for the programmed AV delay. Importantly, this interval varies among manufacturers. Reported safety pacing or crosstalk sensing intervals are approximately 120 ms for Abbott devices, 100 ms for Biotronik devices, 110 ms for Medtronic devices, and 95 ms for Sorin devices, whereas some Boston Scientific devices do not use an equivalent safety pacing feature (9).

### 3.2 Pacing modality in patients with RA reconstruction

For patients with SSS and structurally normal hearts, guidelines favor atrial-based pacing (AAI or DDD) over VVI pacing because atrioventricular synchrony is preserved and unnecessary ventricular pacing can be minimized by programming. Therefore, implantation of a dual-chamber system was considered appropriate in our patient despite the anticipated technical challenges associated with prior RA surgery. However, the optimal pacing strategy may change when lead-related mechanical complications occur. In our patient, a substantial portion of the RA wall had been resected and replaced with a bovine pericardial patch, creating an abnormal anatomical substrate with uncertain long-term lead stability. Under these circumstances, atrial lead revision carried a significant risk of recurrent dislodgement. For this reason, the device was reprogrammed to VVI mode and the atrial lead was extracted. Although long-term ventricular pacing may theoretically increase the risk of pacemaker syndrome and pacing-induced cardiomyopathy, follow-up demonstrated preserved biventricular systolic function, absence of ventricular remodeling, and no evidence of clinically significant pacing-related complications. Therefore, in selected patients with surgically altered RA, a less physiological but mechanically reliable pacing strategy may represent a reasonable alternative when recurrent atrial lead instability is anticipated. Importantly, this decision does not preclude future physiological pacing. Continued surveillance with serial echocardiography and device interrogation has been planned, and if pacing-induced cardiomyopathy or pacemaker syndrome develops, upgrading to physiological pacing remains a reasonable option.

### 3.3 Future perspectives

Several emerging approaches may improve pacing management in patients with reconstructed RA. Alternative atrial pacing sites, including the Bachmann bundle region and interatrial septum, may provide more stable lead fixation and improved atrial activation compared with conventional RA appendage pacing (16–18). A study of atrial lead implantation in myotonic dystrophy patients—a condition associated with atrial myopathy—concluded that the interatrial septum in the region of Bachmann's bundle is the optimal site compared to high lateral RA wall and RA appendage (19). Individualized positioning of the atrial pacing lead guided by mapping of Bachmann's bundle may be one of the new frontiers for atrial pacing (20). In addition, active-fixation leads, including fixed-helix designs and lumenless leads such as the Medtronic SelectSecure™ 3830, offer greater flexibility for implantation at non-conventional atrial locations and show better lead stability (21–23). However, evidence supporting these approaches in patients after RA resection and reconstruction is currently lacking. In reconstructed atria, distorted anatomy, extensive scar formation, and patch material may make localization of the Bachmann bundle difficult and procedural feasibility and long-term electrical performance remain uncertain in this setting.

Another promising development is atrial leadless pacing. The recently introduced Aveir™ AR system enables dedicated atrial pacing without the need for a transvenous atrial lead and therefore eliminates the principal mechanism responsible for lead dislodgement (21, 24). Nevertheless, experience with atrial leadless pacing in patients with reconstructed RA remains extremely limited, and its long-term safety and efficacy require further investigation (25). In our patient, preservation of ventricular systolic function, absence of pacing-related symptoms during follow-up, and the anticipated high risk of recurrent atrial lead failure favored a conservative strategy of atrial lead extraction with VVI pacing.

Taken together, this case emphasizes that successful pacing management after RA reconstruction requires not only accurate diagnosis of lead-related complications but also individualized assessment of the balance between physiological pacing and long-term mechanical reliability.

## 4 Conclusions

This case highlights the diagnostic value of careful ECG interpretation during routine pacemaker follow-up. Atrial lead dislodgement may remain clinically silent yet present with highly characteristic electrocardiographic findings, including atrial pacing–induced ventricular capture and VSP. In patients with surgically reconstructed RA, individualized pacing strategies are required, and single-chamber ventricular pacing may represent a reasonable alternative when recurrent atrial lead instability is anticipated.

## Data Availability

The original contributions presented in the study are included in the article/Supplementary Material, further inquiries can be directed to the corresponding author.
